# Straw Incorporation with Nitrogen Amendment Shapes Bacterial Community Structure in an Iron-Rich Paddy Soil by Altering Nitrogen Reserves

**DOI:** 10.3390/microorganisms9050988

**Published:** 2021-05-03

**Authors:** Juanjuan Wang, Yao Ma, Lin Di, Xiaoqing Qian, Guiliang Wang

**Affiliations:** 1 College of Environmental Science and Engineering, Yangzhou University, Yangzhou 225127, China; wangjuanjuan@yzu.edu.cn (J.W.); mayao1006@outlook.com (Y.M.); qianxq@yzu.edu.cn (X.Q.); 2 Zhenjiang Agricultural Technology Extension Station, Zhenjiang 212009, China; dilin@live.cn

**Keywords:** straw application, nitrogen fertilization, bacterial community, rice paddy

## Abstract

Incorporation of crop straw into the soil along with inorganic fertilization is a widespread agricultural practice and is essential in nutrient-scarce soils, such as iron-rich (ferruginous) paddy soils. The responses of soil bacterial communities to straw incorporation under different nitrogen inputs in iron-rich soils remain unclear. Therefore, 6000 kg ha^−1^ dry wheat (*Triticum aestivum* L. cv. Zhengmai 12) straw was applied to a rice paddy with and without nitrogen amendment (0, 80, 300, and 450 kg ha^−1^ N as urea), to investigate its effects on soil fertility and bacterial community structure. Organic matter, total nitrogen, and water contents tended to decrease in straw-incorporated soils with different nitrogen inputs. Proteobacteria was the dominant bacterial phylum across all treatments (26.3–32.5% of total sequences), followed by Chloroflexi, Acidobacteria, and Nitrospirae. Up to 18.0% of all the taxa in the bacterial communities were associated with iron cycling. Straw incorporation with nitrogen amendment increased the relative abundance of iron oxidizers, Gallionellaceae, while decreasing the relative abundance of iron reducers, Geobacteraceae. Bacterial community composition shifted in different treatments, with total nitrogen, water, and Fe(III) contents being the key drivers. Straw incorporation supplemented by 300 kg ha^−1^ N increased bacterial richness and enhanced all the predicted bacterial functions, so that it is recommended as the optimal nitrogen dosage in practice.

## 1. Introduction

Straw application is a widespread agronomic practice in developed countries [1,2]. However, in China, burning and discarding still account for a large fraction of the disposed straw under an annual straw productivity of 900 million tons [3]. Such improper agronomic practices have not only led to nutrient losses [4] but also to environmental challenges. Open burning of crop straw, for instance, has raised major concerns with regard to air pollution [5]. In recent years, straw application has become increasingly popular in China [6], and numerous studies have investigated the effects of straw application on soil fertility [7,8,9]. Straw application could improve soil structure and water content, as well as soil nitrogen (N) content and N use efficiency [10]. Previous studies have also reported that long-term straw application enhances carbon (C) sequestration considerably, by 50–100%, in China’s subtropical paddy soils [11,12]. According to Potthoff et al. [13], applying straw could increase soil microbial C, and, in turn, soil C stocks, which are already relatively high in Chinese croplands [14,15].

The effects of straw application on soil nutrients are rather complex. Generally, soil organic matter (SOM) is influenced by soil type and biomass, among other factors. Different types of straw have variable C/N ratios, which show equally varying effects on SOM [16]. In addition, SOM content affects soil physicochemical and biological properties, which could control straw decomposition and nutrient cycling [17]. Due to the high C/N ratios of cereal crop straw (up to 80% in wheat straw), the N in straw is mostly retained by microbes during decomposition [18,19]. Studies have shown that straw application without additional N fertilization decreases rice yield, potentially due to competition between straw decomposition and rice growth, which impairs N uptake by plants [20]. Therefore, straw application to the field is usually supplemented by N fertilizer, and N input rate could influence crop productivity. However, few studies have explored the effects of straw application with different N fertilization rates on soil nutrient conditions.

Soil microorganisms balance soil C and other nutrients, and have been demonstrated to facilitate the maintenance of soil health and crop growth [21,22,23]. Soil microbial communities exhibit diverse responses to straw application. According to Tardy et al. [24], soil bacteria exhibit strong responses to wheat straw incorporation, with both species richness and evenness decreasing transiently, regardless of soil history. Changes in soil nutrient status following straw application could be directly associated with microbial processes and could regulate microbial community composition and function [25,26,27]. However, inconsistent results have been reported with regard to the effects of crop straw application on soil bacterial community structure. Specifically, the effects of straw application could vary with different soil types, fertilizer types, straw types, climatic conditions, and agricultural practices [25,28,29]. Consequently, further studies on the effects of straw application on soil bacterial communities are required.

Iron (Fe) is a redox-sensitive metal that is critical for the cycling of soil organic C and other elements. Soil Fe oxides strongly interact with SOM and facilitate SOM stabilization [30]. Periodic wet and dry conditions in paddy soils lead to continual redox reactions of Fe [31], which could be accelerated by diverse microorganisms. For example, Fe(III) reduction is responsible for a large proportion of C mineralization in soils (up to 80%) [32], which is correlated with the presence of Fe(III)-reducing microorganisms. Furthermore, researchers have demonstrated that Fe(III) reducers might compete for electrons with methanogens, leading to decreased methane (CH_4_) emissions in rice paddies [33,34]. In addition, Hu et al. [35] showed that Fe(II) addition decreased CH_4_ emissions induced by rice straw application in flooded paddy soils.

Soil Fe oxides adsorb a wide range of organic anions and cations [36], and the formation of organo-Fe complexes could reduce the microbial degradation of labile organic molecules, such as glucose and citrate [23]. Conversely, organic compounds regulate Fe transformation in soil environments. Adding plant residues could stimulate microbial reduction of native soil Fe oxides and, in turn, influence the associated microbial community structures [37]. Moreover, Fe(II) oxidation and Fe(III) reduction are coupled to N biogeochemical processes in flooded paddy soils [38]. Paddy soils are key habitats for microorganisms involved in Feammox, a major N loss pathway, with potential major bacterial drivers such as Geobacter, Pseudomonas, and Thiobacillus [39,40,41]. Reduced forms of Fe(II) would be oxidized by microaerophilic or nitrate-reducing Fe oxidizers that are ubiquitous in paddy soils, which accelerates the turnover of N [42]. However, it remains unclear how soil bacterial communities respond to straw application supplemented by N fertilizer in paddies with high Fe concentrations.

The Yangtze River Delta in China is a major rice production area. Numerous studies have previously been carried out to explore the effects of straw application on soil fertility and crop yield in this region [43]. However, our understanding of the response of soil bacterial communities to straw application and the optimal N fertilization rate when supplemented with straw is still poor [44], specifically in Fe-rich (ferruginous) paddy soils. Therefore, the objectives of the present study were to investigate the effects of straw incorporation on soil bacterial community structure and to determine the optimal N application rate in combination with straw incorporation for improving soil function in an Fe-rich rice paddy.

## 2. Materials and Methods

### 2.1. Study Site

The field experimental site (31°53′36.70″ N, 119°24′2.81″ E) was located in Zhenjiang, Jiangsu Province, China. This site belongs to the northern subtropical climate zone, with an average annual temperature of 15.6 °C. The average annual precipitation is 1088.3 mm and occurs mostly from June to August. Paddy soil is the main soil type at the experimental site. Due to the influence of irrigation and seasonal precipitation, there are frequent shifts in soil oxidation status, which leads to the high accumulation of Fe in the tillage layer. Fe plaque is also common in rice paddies at the experimental site.

### 2.2. Experimental Design and Soil Sampling

**Figure 1:**
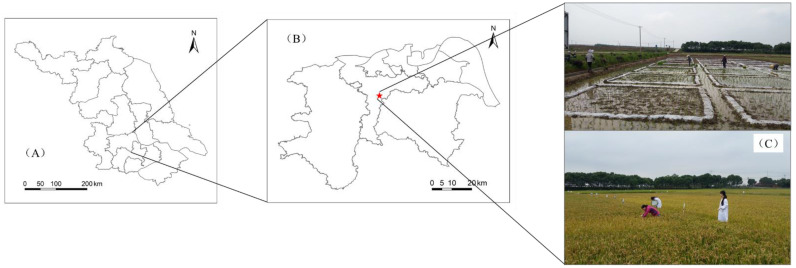
Geographical location of the experimental site. (**A**) Map of Jiangsu Province, China; (**B**) map of Zhenjiang City; (**C**) photos of paddy field.

A rice paddy with an area of 0.1 ha was selected for straw incorporation (Figure 1). A randomized complete block design was adopted for the experiments. Five fertilization treatments with different N rates were applied under wheat straw incorporation (ST0, ST1, ST2, and ST3) or no straw incorporation (T1; Table 1). The wheat straw applied was from the previous season. It contained 460 g kg^−1^ total C, 3.17 g kg^−1^ total N, 0.38 g kg^−1^ total phosphorus (TP), 14.64 g kg^−1^ total potassium (TK), 1.4 g kg^−1^ total sodium, 1.80 g kg^−1^ total calcium, 1.20 g kg^−1^ total magnesium, 1.94 g kg^−1^ total sulfur (S), and 3.20 g kg^−1^ total chlorine. The straw was chopped into small pieces (<2 mm in length) and mixed thoroughly with the surface soil via ploughing. Additionally, N, P, and K were applied as basal fertilizers together with straw (Table 1). Each treatment had three triplicates, yielding 15 experimental plots (30 m^2^). The rice variety under cultivation was *Oryza sativa* subsp. Keng cv. Nanjing 9108. Rice seedlings were transplanted on June 10th and harvested on October 30th. The experiment was repeated each year between 2017 and 2019. The results presented in this paper were observed in the third year (2019).

Soil samples were obtained at the rice ripening stage, right before draining the field. A minimum of 10 soil cores (5 cm in diameter and 15 cm in depth) from each plot were obtained randomly. The topsoil (0–2 cm) was removed to minimize the potential influence of debris. The samples obtained from one plot were pooled, mixed uniformly, and then divided into two portions. One portion was transported on ice to the laboratory and frozen immediately at −80 °C for use in subsequent DNA extraction and sequencing analyses, and the other portion was air-dried for the determination of soil physicochemical properties. The above procedures were repeated for each plot and a total of 15 samples were taken.

### 2.3. Soil Physicochemical Analysis

Soil samples were air-dried and sieved with 0.85 mm and 0.15 mm sieves for use in the determination of pH and other properties, respectively. Deionized water and soil were mixed at a ratio of 2.5:1 (*v*/*w*) and vortexed; soil pH and electrical conductivity (EC) were determined using a digital pH meter (Sanxin S731; Shanghai San-Xin Instruments, Shanghai, China). Gravimetric water content (WC) was measured as the difference in WC between the moist soil and the soil dried at 105 °C for 24 h. SOC, TN, and TP contents were determined according to standard testing procedures [45]. Briefly, SOC was determined using the potassium dichromate volumetric-external heating method; TN was determined using the Kjeldahl method; TP was determined using the HClO_4_-H_2_SO_4_ extraction–Mo-Sb anti-spectrophotometric method. Fe(II) and Fe(III) contents were determined using the 0.5 N HCl extraction and phenathroline spectrophotometric method [46]. Non-crystal Fe oxide content was determined using the dithionite-citrate-bicarbonate (DCB) extraction technique and phenathroline method [47].

### 2.4. DNA Extraction, PCR Amplification, and Illumina MiSeq Sequencing

Genomic DNA was extracted from 0.5 g freeze-dried soil samples using the MoBio PowerSoil DNA Isolation Kit (QIAGEN Inc., Valencia, CA, USA) according to the manufacturer’s protocols. The quality and quantity of the DNA samples were checked using a spectrophotometer (Nano Drop ND2000; Thermo Scientific, Wilmington, DE, USA).

To amplify the hypervariable V3–V4 regions of the 16S rRNA gene, the bacterial primer set 338F (5′-ACTCCTACGGGAGGCAGCAG-3′) and 806R (5′-GGACTACHVGGGTWTCTAAT-3′) was used [48]. A PCR reaction contained a 25 μL mixture with 10 μL MilliQ water, 5 μL 5 × FastPfu Buffer, 2 μL 2.5 mM dNTPs, 1.0 μL of primer 338F (5 μM), 1.0 μL of primer 806R (5 μM), 0.5 μL FastPfu Polymerase, 10 ng template DNA, and 0.25 μL bovine serum albumin. The amplification conditions were as follows: DNA denaturation at 94 °C for 3 min, followed by 30 cycles of amplification (94 °C for 45 s, 50 °C for 45 s, and 72 °C for 45 s), and final extension for 10 min at 72° C. PCR reactions were performed in triplicate. The PCR products were gel-purified using an AxyPrepDNA Gel Extraction Kit (Axygen Biosciences, Union City, CA, USA) and quantified using QuantiFluor™-ST (Promega, Madison, WI, USA) according to the manufacturers’ instructions. Sequencing of the purified PCR products was conducted on an Illumina MiSeq platform (Illumina, San Diego, CA, USA) by Majorbio Bio-Pharm Technology Co. Ltd. (Shanghai, China) according to standard protocols, with 250-bp paired-end reads generated.

The sequences obtained were analyzed using Mothur v1.30.2 [49]. Raw sequences were assigned to samples according to the barcodes, and those that did not perfectly match the primer/barcode or were <250 bp in length were removed. Chimeras were removed using the UCHIME algorithm with default parameters implemented in Mothur. The unique sequences were merged, chopped to achieve similar lengths, and then aligned against the SILVA132 16S rRNA database (https://www.arb-silva.de; accessed on 2 September 2020) on the Mothur platform to obtain taxonomic information on bacterial communities. Non-bacterial reads were further removed. Operational taxonomic units (OTUs) were clustered based on 97% similarity [50]. The number of sequences in each sample was normalized to the minimum number of sequences per sample. Bacterial alpha-diversity was estimated using the observed species (Sobs), ACE, and Shannon indexes generated based on the OTU counts.

### 2.5. Data Analysis

Bioinformatics analysis of bacterial sequence data was performed using a free online platform, Majorbio I-Sanger Cloud (http://www.i-sanger.com; accessed on 5 February 2021). One-way analysis of variance (ANOVA) followed by the Tukey–Kramer test was performed to investigate differences in soil physiochemical properties, bacterial alpha-diversity indexes, and dominant taxa abundances among treatments, with *p* < 0.05 considered to indicate significant difference. Non-metric multidimensional scaling (NMDS) analysis based on the Bray–Curtis distances was performed on bacterial community composition at both the phylum and genus levels, with the significance of differences tested by the analysis of similarities (ANOSIM). The influences of environmental factors on bacterial community structure were estimated using redundancy analysis (RDA). The correlations between abundant bacterial taxa (the top 15 phyla and 30 genera) and soil properties were calculated based on Spearman’s correlation. Functional genes and metabolic pathways were predicted with Phylogenetic Investigation of Communities by Re-construction of Unobserved States (PICRUSt v1.1.0; https://github.com/picrust; accessed on 8 February 2021).

## 3. Results

### 3.1. Soil Physicochemical Properties

The soil samples were acidic, with an average pH of 5.05 (Table 2). There was no statistically significant difference in soil pH among the treatments. However, the ST1 treatment with straw and low N had the highest EC, which was significantly higher than that of the T1 treatment with low N alone (*p* < 0.05). In contrast, the highest SOM (32.29 g kg^−1^), TP (0.60 g kg^−1^), TN (1.75 g kg^−1^), and WC (65.94%) contents were observed in the T1 treatment. Compared with T1, straw incorporation without N (ST0) and with low N (ST1) decreased SOM; surprisingly, straw incorporation with N (ST1 to ST3) decreased TN and WC (*p* < 0.05).

The rice paddy was rich in Fe and rust-colored Fe oxides were commonly observed in soil cores. The dynamics of soil Fe content were rather complex (Table 1). The bioavailable Fe and non-crystal Fe oxide contents were not significantly influenced by the different treatments. However, Fe(II) content was markedly low in the ST2 treatment, and Fe(III) content was relatively low in the ST0, ST1, and ST2 treatments, whereas the lowest DCB-Fe content was observed in the T1 treatment.

### 3.2. Soil Bacterial Community Diversity

The calculated alpha-diversity indexes of bacterial communities in soil samples associated with different treatments are listed in Table 3. The Sobs and ACE indexes represent bacterial species richness, while the Shannon index represents bacterial species evenness; higher values of these indexes suggest greater bacterial richness and lower heterogeneity, respectively. Overall, the coverage between the samples was similar, ranging from 95.9% to 97.2%, indicating that the sequences were representative. The ST2 treatment had the highest Sobs and ACE index values. The highest Shannon index values were observed in the T1 treatment, and they were significantly higher than those in ST3 treatments (*p* < 0.05). Generally, straw incorporation alone or with low N decreased bacterial richness slightly; straw incorporation with moderate N increased bacterial richness but decreased evenness, whereas straw incorporation with high N decreased both bacterial richness and evenness.

NMDS analysis was performed to visualize variations in bacterial community composition among the treatments (Figure S1). A shift in bacterial community composition was observed following straw incorporation and the bacterial community in T1 samples was clearly separated from the remaining treatment groups at both the phylum and genus levels. An increase in N application also induced a shift in bacterial community composition under straw incorporation, with ST3 forming an individual cluster and clearly separating from the communities in ST0, ST1, and ST2 samples, which were not entirely separate. Furthermore, ANOSIM test results revealed that the bacterial community compositions in T1 and ST3 were significantly different (*p* < 0.05) from those of the remaining treatment groups.

### 3.3. Taxonomic Composition of Bacterial Communities

**Figure 2:**
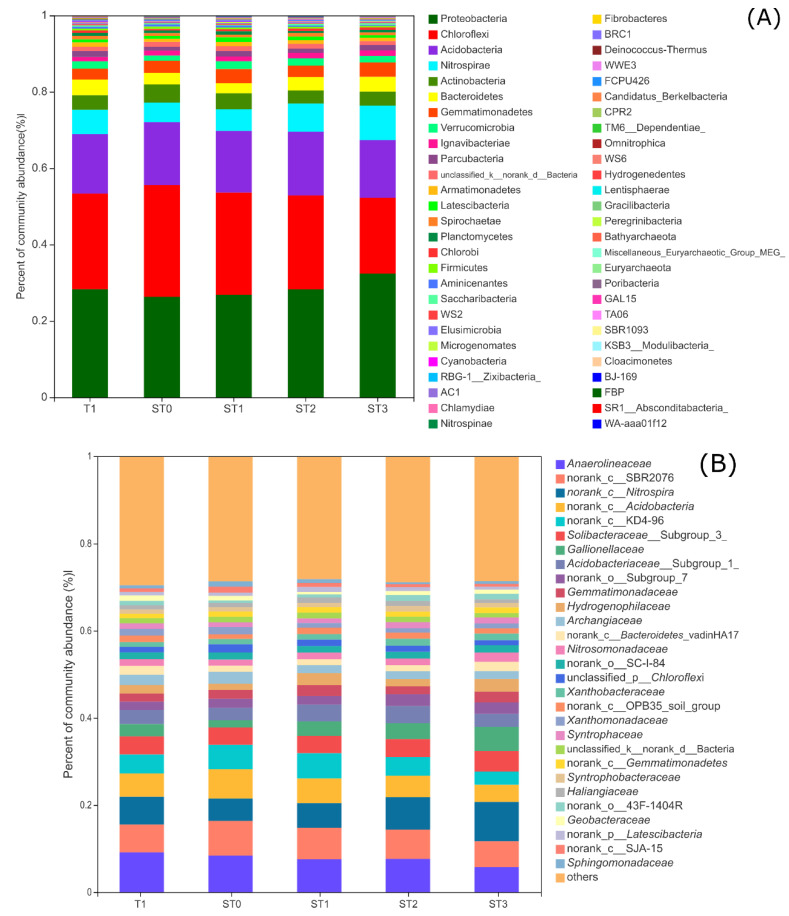
Relative abundances of major bacterial taxa present at the phylum (**A**) and family (**B**) levels in paddy soils under different treatments. Only phylogenetic groups represented by more than 1% of the total reads at the family level are shown. T1, nitrogen fertilization alone (80 kg ha^−1^); ST0, straw incorporation alone (6000 kg ha^−1^); ST1, straw incorporation with low nitrogen (80 kg ha^−1^); ST2, straw incorporation with moderate nitrogen (300 kg ha^−1^); ST3, straw incorporation with high nitrogen (450 kg ha^−1^).

Based on the taxonomic classification of 16S rRNA sequences, 54 bacterial phyla were recognized across all samples. Figure 2 presents the taxonomic assignments of the sequences at the phylum or family level. Proteobacteria was the most dominant phylum in all samples and accounted for 28.5% of the total sequences (Figure 2A). The second most abundant phylum was Chloroflexi (25.1%), followed by Acidobacteria (16.0%), Nitrospirae (6.72%), Actinobacteria (3.98%), Bacteroidetes (3.41%), and Gemmatimonadetes (3.32%). These seven dominant phyla accounted for 87.0% of the total bacterial community.

Proteobacteria had the highest relative abundance in the ST3 treatment (32.5%) of the total sequences, and its lowest relative abundance was observed in the ST0 treatment (26.3%). Within the phylum Proteobacteria, the majority (72%) of the sequences belonged to the classes Betaproteobacteria and Detaproteobacteria. Alphaproteobacteria and Gammaproteobacteria accounted for 17.9% and 8.48% of the Proteobacterial sequences, respectively. The distribution patterns of Chloroflexi were opposite to those of Proteobacteria, with the highest relative abundance observed in the ST0 treatment (29.3%) and the lowest in the ST3 treatment (19.8%). The relative abundances of the dominant phyla varied somewhat among the treatments; however, significant differences were observed only in the less dominant phyla, including Verrucomicrobia, Ignavibacteriae, Planctomycetes, AC1, and Peregrinibacteria (*p* < 0.05; Figure S2A). The highest relative abundances of Verrucomicrobia and Planctomycetes were observed in the ST1 treatment, while the relative abundance of Ignavibacteriae was the highest in the ST3 treatment.

Bacterial families with relative abundances > 3% included Anaerolineaceae, norank_c_SBR2076, norank_c_Nitrospira, norank_c_Acidobacteria, norank_c_KD4-96, Solibacteraceae_Subgroup_3, Gallionellaceae, and Acdiobacteriaceae_Subgroup_1 (Figure 2B). The top two dominant families belonged to the phylum Chloroflexi. Among the dominant families, the relative abundance of Anaerolineaceae was the highest in the T1 treatment (7.7%). Norank_c_Nitrospira and Gallionellaceae had their highest relative abundances in the ST3 treatment (9.02% and 5.56%, respectively). In addition, the relative abundances of unclassified norank_c_SBR2076 and norank_c_KD4-96, belonging to the phylum Chloroflexi, were significantly lower in ST3 than in ST0 and ST1 treatments, respectively (*p* < 0.05).

More than 18.0% of the total sequences belonged to Fe-cycling-related bacteria. The relative abundance of Gallionellaceae, a typical Fe(II)-oxidizing bacterial group, was significantly higher in the ST3 treatment than in the ST0 treatment (*p* < 0.05). The relative abundance of Geobacteraceae, a family containing many Fe(III)-reducing bacteria, was significantly higher in the T1 treatment than in the ST1 treatment (*p* < 0.05; Figure S2B).

### 3.4. Influence of Environmental Factors on Bacterial Community Structure

**Figure 3:**
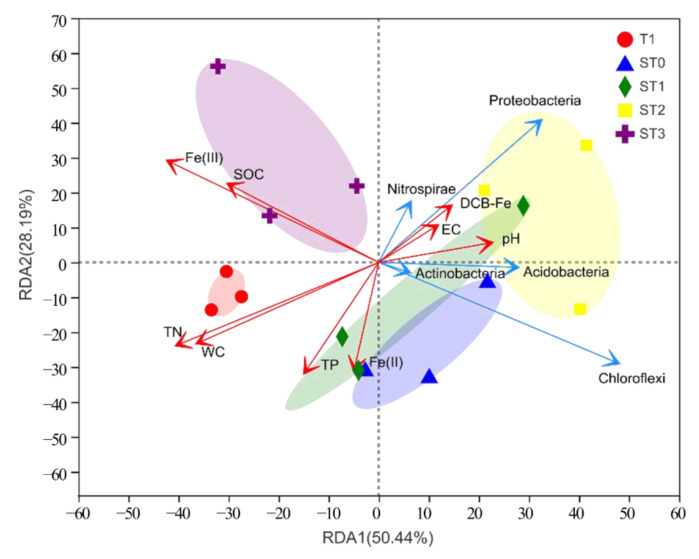
Results of redundancy analysis of bacterial community structure (OTU assignment: distance cutoff 0.03) retrieved from Illumina sequencing of the 16S rRNA genes in relation to soil properties and depth level. SOC: soil organic carbon; EC: electrical conductivity; TN: total nitrogen; TP: total phosphorus; WC: water content; Fe(II): ferrous iron; Fe(III): ferric iron; DCB-Fe: dithionite-citrate-bicarbonate-extracted iron. Treatment abbreviations are defined in Figure 2 legend.

The relationships between the distribution of the major bacterial groups in rice paddy soils and the soil physicochemical properties in different treatments were analyzed by RDA (Figure 3). The first two axes explained 78.13% of the total variance in the bacterial community (RDA1 = 50.44% and RDA2 = 28.19%) at the phylum level. The bacterial community structure differed among the treatments, with T1 and ST3 samples clearly separated from the remaining treatment groups, suggesting that there were distinct geochemical processes influencing bacterial community structure in the two treatments. Bacterial community compositions in ST0, ST1, and ST2 samples were relatively closely related. Soil TN content (r^2^ = 0.53, *p* = 0.015), water content (r^2^ = 0.44, *p* = 0.03), and Fe(III) content (r^2^ = 0.66, *p* = 0.004) were the key environmental factors influencing the bacterial community composition. Soil bioavailable Fe(III) and SOC contents shaped bacterial community structure in the ST3 treatment, while soil pH and DCB-Fe content were more associated with the bacterial community composition in the ST2 treatment.

To further reveal the influence of environmental factors on soil bacterial community structure, the relationships between individual bacterial groups and soil properties were analyzed using Spearman correlation coefficients (Table 4). The relative abundances of Proteobacteria, Chloroflexi, Acidobacteria, Actinobacteria, Gemmatimonadetes, Ignavibacteriae, unclassified_k_norank_d_Bacteria, and Spirochaetae were negatively correlated with soil TN content (*p* < 0.05). The relative abundances of Chloroflexi, Actinobacteria, Gemmatimonadetes, and Spirochaetae were negatively correlated with SOM content (*p* < 0.05). In addition, the relative abundance of unclassified_k_norank_d_Bacteria was negatively correlated with soil Fe(II) content (*p* < 0.05), and the relative abundance of Spirochaetae was negatively correlated with soil DCB-Fe content (*p* < 0.05). The relative abundances of Chloroflexi and Actinobacteria were negatively correlated with soil Fe(III) content, whereas the relative abundance of Verrucomicrobia was positively correlated with this soil property (*p* < 0.05).

### 3.5. Predicted Functions of Bacterial Communities

**Figure 4:**
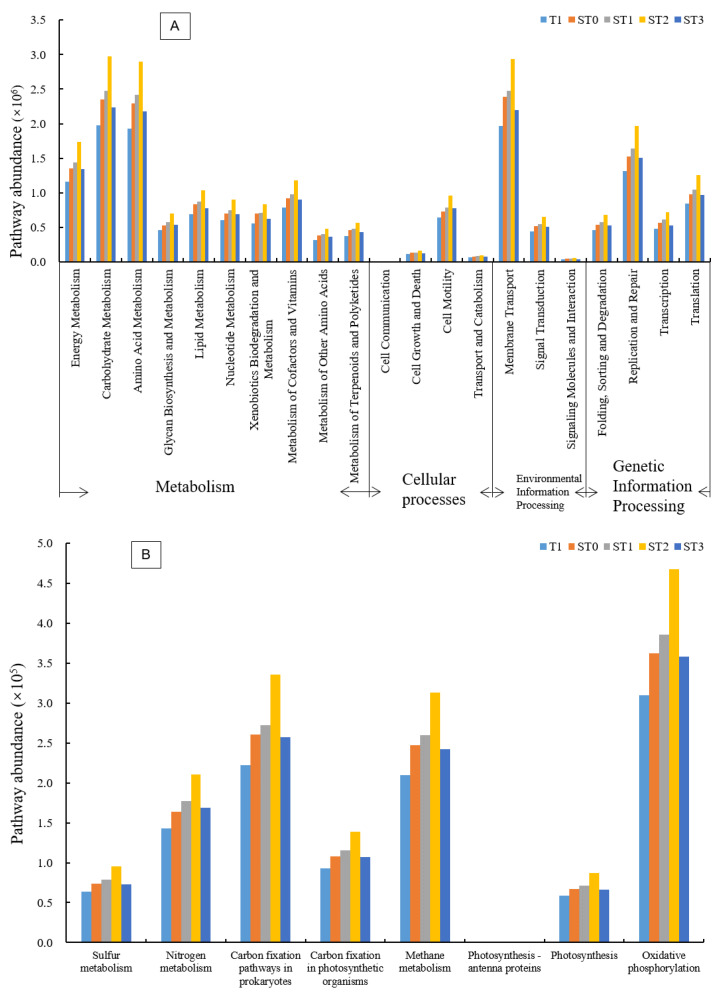
Kyoto Encyclopedia Genes and Genomes-predicted metabolic pathways (**A**) and energy metabolism pathways (**B**) of soil bacterial communities in different treatments. Treatment abbreviations are defined in Figure 2 legend.

PICRUSt analysis was performed to predict the potential functions of soil bacterial communities in the different treatments (Figure 4). The abundances of major functional pathways, including metabolism, cellular processes, environmental information processing, and genetic information processing, were compared among the treatments. Notably, the ST2 treatment had the highest abundances for all functional pathways, followed by ST1 and ST0 treatments. The T1 treatment had the lowest abundance of all functional pathways (Figure 4A). Further analyses on the energy metabolism pathways revealed that the predicted pathways associated with C fixation, methane production, and oxidative phosphorylation were relatively more abundant (Figure 4B). In addition, the ST2 treatment had the highest abundances of pathways associated with S, N, and CH_4_ metabolism, C fixation, photosynthesis, and oxidative phosphorylation. Overall, straw incorporation with N amendment was beneficial for cell growth and metabolism, and the positive effect first increased with increasing N input, and then dropped when a high N rate (450 kg ha^−1^) was applied.

## 4. Discussion

### 4.1. Effects of Straw Incorporation with N Amendment on Paddy Soil Fertility

Straw application has a compound effect on soil nutrient status. On one hand, it could increase SOM [20,25] and stimulate soil microbial growth and activities [44], which, in turn, accelerate the release of diverse nutrients. Conversely, excess C input could cause N immobilization through microbial activities, and, in turn, competition for N between plants and microbes [51]. However, according to our results, straw incorporation did not increase SOM content in the rice paddy, which is inconsistent with the findings of many previous studies [11,12,13,14,15]. One of the reasons could be that the ameliorating effects of straw incorporation occurred in the short term and microbial and plant growth took up much more organic matter in the long term. This might even be associated with SOM decomposition stimulated by straw input, which could explain the highest SOM content in the T1 treatment that had no straw incorporation. Similarly, strong regional variation in the effects of straw application on SOC stocks, and a lack of increase in SOC stocks in regions with low SOC densities have been reported [52]. The decrease in soil TN content with straw incorporation could be attributed to straw-derived increase in N use efficiency and an increase in N loss under high N input [53,54].

Compared with the T1, soil WC dropped following straw incorporation, which could be due to a change in soil structure because of straw decomposition and enhanced bacterial activity. In a previous study, straw application accompanied with moderate N (225 kg ha^−1^) achieved the maximum grain yield of winter wheat with the improvement of soil structure [55]. Here, we observed that N input eventually decreased soil TN content, and the optimal N application rate, which resulted in the highest rice yield (Supplementary Table S2), could be ~300 kg ha^−1^. Rice yield decreased in the ST3 treatment, which had the highest input, suggesting that the optimal N application rate in the ST3 treatment should not be exceeded.

### 4.2. Effects of Straw Incorporation with N Amendment on Bacterial Community Structure

Soil bacterial communities regulate the biogeochemical processes of soil elements, while crop straw provides energy and nutrients required for the growth of soil bacteria [23]. Previous research has shown that straw application influences soil bacterial community composition profoundly [25]. Straw application not only provides rich nutrients but also improves soil structure, thereby increasing soil bacterial diversity [56]. In the present study, however, straw incorporation alone or with low N (80 kg ha^−1^) decreased soil bacterial richness slightly; in contrast, straw incorporation amended with moderate N (300 kg ha^−1^) increased bacterial richness, which mildly decreased again when N amendment was increased to a high rate (450 kg ha^−1^). High N could stimulate the growth of less abundant copiotrophic bacterial groups that are K strategists, and thus decrease the relative abundances of other dominant bacterial groups [57,58] despite an increase in C input [59,60]. The lowest bacterial diversity index values were observed in the ST3 treatment, which had the highest N input. This is probably due to the facilitation of major bacterial groups following an increase in N input.

Across all treatments, Proteobacteria was the most dominant bacterial phylum, followed by Chloroflexi, Acidobacteria, and Nitrospirae, which is consistent with the findings of previous studies in paddy soils [61,62]. Proteobacteria is composed of numerous classes that are sensitive to copiotrophic conditions (K strategy), such as Beta- and Delta-proteobacteria [63]. In the present study, Beta- and Delta-proteobacteria were only slightly more abundant in the ST2 and ST3 treatment when compared with the other treatments, indicating that only with moderate to high N supplementation could straw incorporation increase the abundance of such classes of Proteobacteria. Many taxa of this phylum are known to promote plant growth via improving soil functions and regulating plant immune systems [64], and thus contribute to rice production.

Only a small proportion of the soil microbial populations could benefit from straw incorporation as a C source either directly or indirectly as predators of primary degraders [65,66]. Straw breakdown requires the activities of various microbial groups [67] and low soil pH, which would alter the nutrient status for bacterial communities. Nevertheless, soil pH exhibited minimal change following straw incorporation in the present study. High N input decreased the relative abundances of several potential Fe(III)-reducing groups, including norank_c_SBR2076 and norank_c_KD4-96 of the phylum Chloroflexi, in addition to norank_c_Acidobacteria. Meanwhile, high N input increased the relative abundance of an Fe(II)-oxidizing group, Gallionellaceae, and a potentially keystone group, nonrank_c_Nitrospira with functions in Fe uptake and nitrite oxidation [68]. This could be related to the enhanced plant growth that increased the radial oxygen (O_2_) loss favorable for Fe(II)-oxidizing bacteria.

### 4.3. Relationship between Bacterial Community Structure and Soil Environment

Microorganisms enhance soil functions and productivity through their participation in nutrient cycling and organic matter turnover [69]. Conversely, soil environmental factors influence the community composition of soil microorganisms. In the present study, we observed that soil bacterial community composition in the samples without straw was distinct from that of samples with straw incorporation. Extra nutrients introduced by straw incorporation could have altered bacterial community composition. Bu et al. [28] also showed that soil bacterial communities were separated into two groups between a straw-incorporated treatment and non-straw-incorporated treatment in a rice–rice–rapeseed rotation system. Among the treatments with straw incorporation, the bacterial community composition in ST3 samples was distinct from that of the others, demonstrating that high N input had strong effects on soil bacterial community composition [57,59].

In this study, the paddy soil had very high Fe contents and rust-colored Fe oxides were common in the field. The rapid Fe cycling associated with straw decomposition in the Fe-rich paddy soil could be driven by bacterial communities. According to our RDA results, Fe(III) content was a key environmental factor, similar to TN content, influencing soil bacterial community structure. A previous study also showed that Fe oxides could influence soil bacterial community structure in arsenic-contaminated soils [70]. Therefore, we specifically explored the bacterial groups associated with Fe cycling.

Not surprisingly, Gallionellaceae, widely recognized Fe(II)-oxidizing bacteria, were abundant in all samples. Gallionellaceae are microaerophilic bacteria that grow chemoautotrophically using Fe(II) as an energy source and carbon dioxide as a C source under low O_2_ conditions, which are particularly important traits at the oxic–anoxic interfaces of Fe-rich environments [71]. According to the results of Spearman correlation analysis, *Gallionella* abundance was positively correlated with the content of non-crystal Fe oxides (Table S1). In addition, straw incorporation with N amendment increased the relative abundance of Gallionellaceae while decreasing the relative abundance of Geobacteraceae; these variations were probably caused by the decrease in soil WC after treatment.

Considering bacterial functions, the ST2 treatment had the highest abundances of pathways associated with S, N, and CH_4_ metabolism, C fixation, photosynthesis, and oxidative phosphorylation. Although there were no clear trends with regard to the effects of straw incorporation on soil bacterial community structure under different N inputs, straw incorporation with N amendment enhanced cell growth and metabolism, with ST2 (straw incorporation + 300 kg ha^−1^ N) being the optimal treatment. Many studies have explored the effects of straw application on the responses to total soil microbial biomass and community structure, with less attention directed at soil microbial diversity and function [58]. Therefore, future studies should focus not only on the effects of straw application on phylogenetic diversity of bacteria but also functional diversity of bacteria, to enhance our understanding of soil ecosystem function following straw application.

## 5. Conclusions

The results of the present study echo the observation that straw incorporation has complex effects on soil nutrient status and bacterial community structure in Fe-rich paddies. Straw incorporation, with application, decreased SOM, TN, and WC contents, without affecting pH and Fe bioavailability. The relative abundances of dominant bacterial phyla, including Proteobacteria, Chloroflexi, Acidobacteria, Nitrospirae, and Actinobacteria, were all negatively correlated with soil TN, TP, Fe(II), and WC contents. The bacterial communities comprised a large proportion of Fe cycling-related bacteria and responded distinctively to different treatments. Soil TN, WC, and Fe(III) contents were the key environmental factors driving the variation in bacterial community structure. In general, straw incorporation with N amendment at a moderate rate (300 kg ha^−1^) most effectively improved rice yield, increased bacterial richness, and enriched the predicted functional pathways associated with cell growth and metabolism. Therefore, 300 kg ha^−1^ N is identified and recommended as the optimal N application rate for supplementing straw incorporation in rice paddies.

## Figures and Tables

**Table 1:** Fertilization treatments applied in the experiments (unit: kg ha^−1^).

Treatments	Wheat Straw	Nitrogen in the Form of Urea	Phosphorus in the Form of Calcium Superphosphate	Potassium in the Form Potassium Chloride
T1	0	80	80	80
ST0	6000	0	80	80
ST1	6000	80	80	80
ST2	6000	300	80	80
ST3	6000	450 *	80	80

* Local farmer practice.

**Table 2:** Summary of the major soil physicochemical properties in different treatments.

Treatments	pH	EC	SOM (g kg^−1^)	TP (g kg^−1^)	TN (g kg^−1^)	WC (%)	Fe(II) (g kg^−1^)	Fe(III) (g kg^−1^)	DCB-Fe (g kg^−1^)
T1	5.05 ± 0.08 a	154.00 ± 7.00 b	32.29 ± 2.61 a	0.60 ± 0.03 a	1.75 ± 0.15 a	65.94 ± 9.31 a	3.81 ± 0.17 a	13.12 ± 1.34 a	74.38 ± 1.08 a
ST0	5.06 ± 0.02 a	164.67 ± 23.01 ab	27.13 ± 1.79 b	0.58 ± 0.26 a	1.57 ± 0.26 ab	58.16 ± 9.51 ab	3.95 ± 1.66 a	10.39 ± 1.58 a	79.24 ± 7.96 a
ST1	5.11 ± 0.22 a	188.33 ± 16.07 a	27.13 ± 0.40 b	0.57 ± 0.04 a	1.46 ± 0.07 b	47.71 ± 1.95 b	3.53 ± 1.28 a	11.84 ± 3.49 a	77.18 ± 3.08 a
ST2	5.05 ± 0.09 a	158.00 ± 14.11 ab	30.21 ± 1.71 ab	0.57 ± 0.02 a	1.40 ± 0.01 b	45.95 ± 4.36 b	2.57 ± 0.17 a	10.53 ± 3.17 a	78.47 ± 11.07 a
ST3	5.00 ± 0.15 a	179.67 ± 19.09ab	30.99 ± 2.86 ab	0.55 ± 0.00 a	1.47 ± 0.08 b	50.84 ± 6.84 b	4.86 ± 1.89 a	14.17 ± 2.34 a	77.92 ± 4.44 a

EC: electrical conductivity; SOC: soil organic matter; TP: total phosphorus; TN: total nitrogen; WC: gravimetric water content; DCB-Fe: dithionite-citrate-bicarbonate-extracted iron. Treatment abbreviations are defined in Table 1. Different letters in the same column indicate statistically significant differences among the samples by one-way ANOVA (Tukey–Kramer, *p* < 0.05).

**Table 3:** Alpha-diversity and richness estimates of soil microbial communities based on 97% similarity OTU clusters.

Treatments	Sobs	ACE	Shannon	Coverage
T1	2841.3 ± 96.03 b	3812.9 ± 47.71 ab	6.86 ± 0.02 a	0.959
ST0	2726.0 ± 85.58 ab	3759.6 ± 188.9 a	6.85 ± 0.07 ab	0.966
ST1	2771.3 ± 101.53 ab	3712 ± 99.25 ab	6.79 ± 0.04 ab	0.968
ST2	2970.7 ± 100.55 a	3843.8 ± 99.4 a	6.81 ± 0.14 ab	0.972
ST3	2667.0 ± 74.73 b	3620.2 ± 39.83 b	6.66 ± 0.09 b	0.964

Treatment abbreviations are defined in Table 1. Different letters in the same column indicate statistically significant differences among the samples based on one-way ANOVA (Tukey–Kramer, *p* < 0.05).

**Table 4:** Spearman correlation coefficients between the relative abundances of dominant bacterial phyla and soil physiochemical properties.

Phylum	pH	EC	SOC	TP	TN	WC	Fe(II)	Fe(III)	DCB-Fe
Proteobacteria	0.24	0.21	0.00	−0.63 *	−0.78 ***	−0.58 *	−0.46	−0.23	0.14
Chloroflexi	0.24	0.06	−0.54 *	−0.02	−0.57 *	−0.31	−0.48	−0.71 **	−0.19
Acidobacteria	0.12	0.15	−0.33	−0.04	−0.70 **	−0.49	−0.51	−0.32	−0.03
Nitrospirae	−0.05	0.30	0.20	−0.33	−0.40	−0.46	−0.41	0.10	0.44
Actinobacteria	0.44	0.07	−0.61 *	−0.29	−0.61 *	−0.35	−0.36	−0.85 ***	−0.07
Bacteroidetes	−0.31	−0.36	0.51	−0.40	−0.27	0.19	−0.14	0.07	−0.12
Gemmatimonadetes	0.42	0.50	−0.62 *	−0.45	−0.74 **	−0.80 ***	−0.24	−0.41	0.20
Verrucomicrobia	−0.53 *	0.25	0.08	0.39	0.09	−0.08	0.00	0.69 **	0.39
Ignavibacteriae	0.14	0.13	−0.10	−0.57 *	−0.80 ***	−0.36	−0.33	−0.12	0.05
Parcubacteria	0.17	0.48 *	−0.28	0.11	−0.31	−0.43	−0.04	0.32	0.46
Unclassified_k_norank_d_Bacteria	0.15	0.50 *	−0.63	0.01	−0.63 *	−0.63 *	−0.66 **	−0.30	0.28
Armatimonadetes	−0.04	0.28	−0.11	0.45	−0.03	−0.26	−0.03	0.43	0.33
Latescibacteria	0.32	0.58 *	−0.46	0.11	−0.29	−0.58 *	−0.50	−0.13	0.14
Spirochaetae	0.18	−0.34	0.04	−0.54 *	−0.52 *	0.02	−0.27	−0.44	−0.55 *
Planctomycetes	0.03	0.54 *	−0.54 *	0.13	−0.32	−0.41	0.10	0.27	0.21

EC: electrical conductivity; SOC: soil organic carbon; TP: total phosphorus; TN: total nitrogen; WC: gravimetric water content; Fe(II), ferrous iron; Fe(III), ferric iron; DCB-Fe: dithionite-citrate-bicarbonate-extracted iron. Asterisks indicate significant difference (* 0.01 < *p* ≤ 0.05, ** 0.001 < *p* ≤ 0.01, *** *p* ≤ 0.001).

## Data Availability

The data presented in this study are openly available in the NCBI Sequence Read Archive, with the accession number PRJNA726334.

## Supplementary Materials

The following are available online at https://www.mdpi.com/article/10.3390/microorganisms9050988/s1, Figure S1: Non-metric multidimensional scaling analysis (NMDS) based on the relative abundance of microbial phylum (A) and genus showing different microbial community structure, Figure S2: The composition of bacteria community in different treatments at phylum (A), family (B) and genus (C) levels, Table S1: Spearman correlation analysis between soil physiochemical parameters and the relative abundance of genus, Table S2: Rice yield components in different treatments.
