# Supplementary material for: Straw Incorporation with Nitrogen Amendment Shapes Bacterial Community Structure in an Iron-Rich Paddy Soil by Altering Nitrogen Reserves
Source: Microorganisms. 2021 May 3;9(5):988. doi: 10.3390/microorganisms9050988 (PMC8147819; doi:10.3390/microorganisms9050988)
Supplement: Supplementary file 1 [file microorganisms-09-00988-s001.zip › microorganisms-1156324-supplementary.pdf]

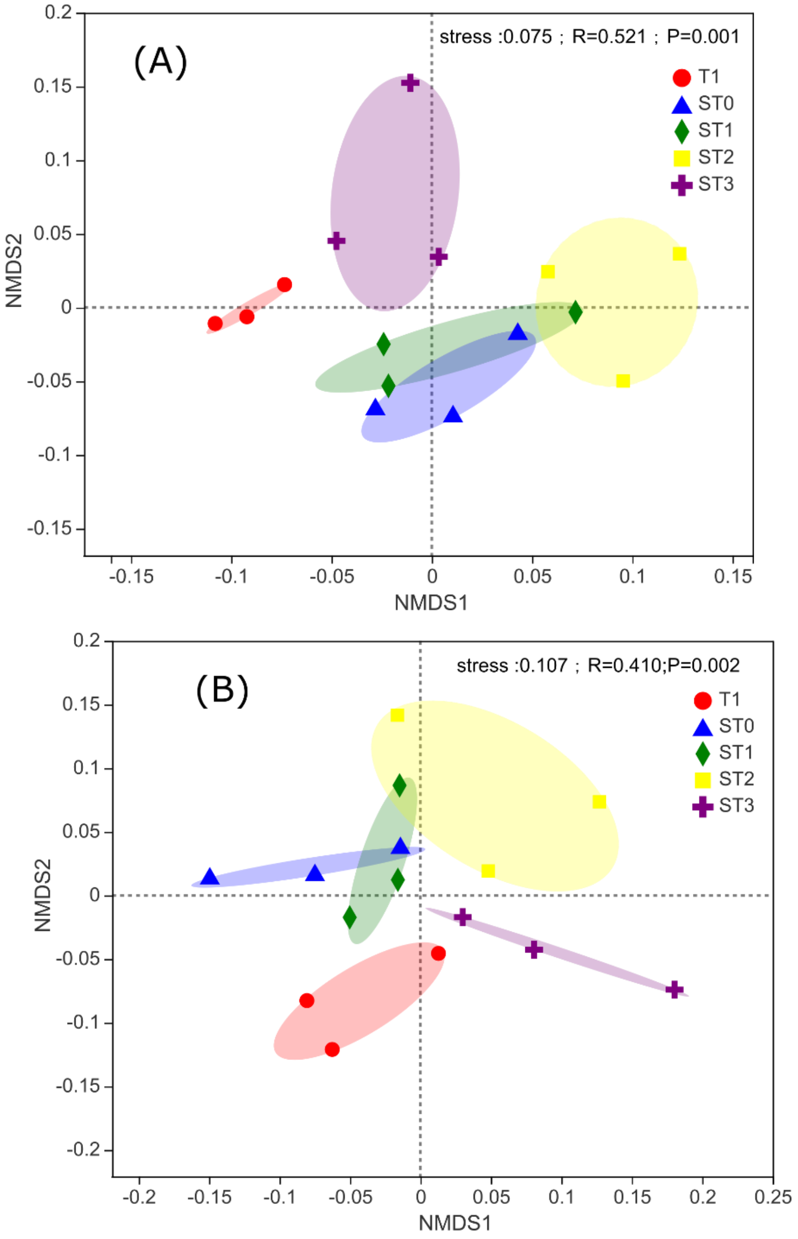

Figure S1 Non-metric multidimensional scaling analysis (NMDS) based on the relative abundance of microbial phylum (A) and genus showing different microbial community structure.

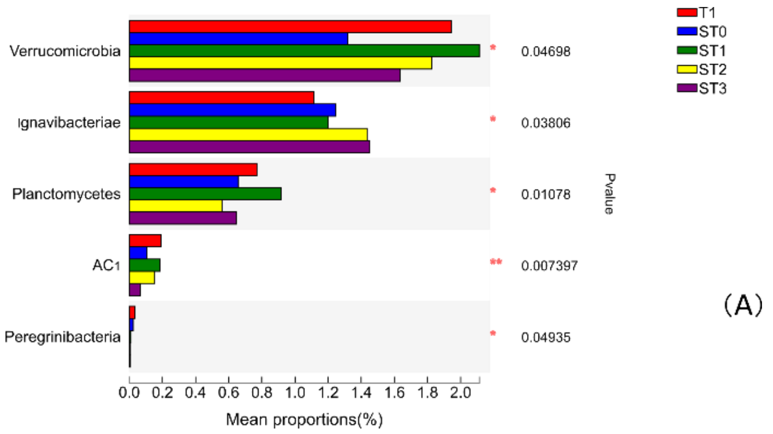

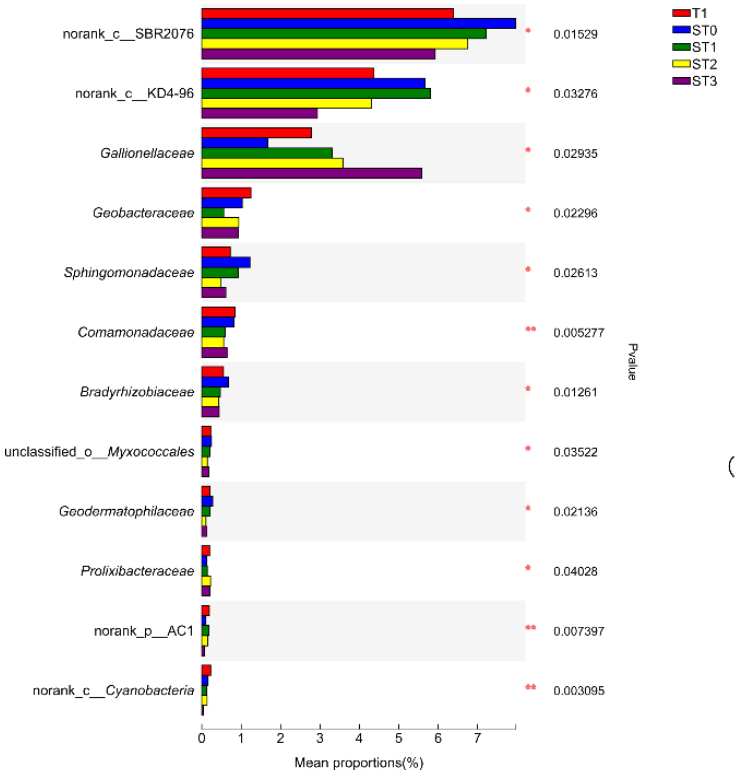

(B)

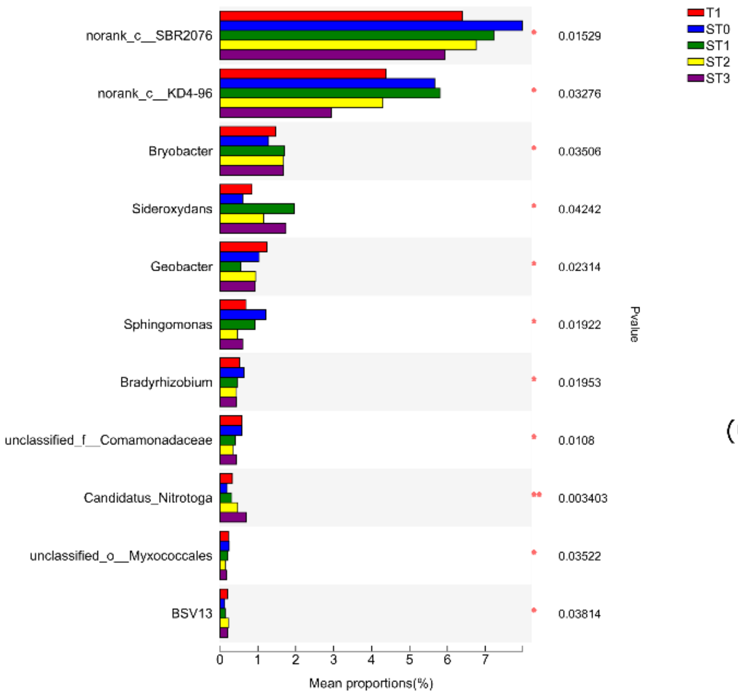

(C)

Figure S2 The composition of bacteria community in different treatments at phylum (A), family (B) and genus (C) levels.

Table S1 Spearman correlation analysis between soil physiochemical parameters and the relative abundance of genus

Bacterial genus	pH	EC	SOC	TP	TN	WC	Fe(II)	Fe(III)	DCB-Fe
g__norank_c__SBR2076	0.24	0.39	**-0.58***	0.05	**-0.63***	**-0.55***	**-0.58***	-0.50	0.09
g__*Nitrospira*	-0.05	0.30	0.20	-0.33	-0.40	-0.46	-0.41	0.10	0.44
g__norank_f__*Anaerolineaceae*	-0.24	-0.48	0.07	0.22	-0.17	0.10	-0.25	-0.28	-0.29
g__norank_c__*Acidobacteria*	**0.52***	**0.06****	-0.64	-0.06	-0.49	-0.32	-0.14	**-0.59***	-0.31
g__norank_c__KD4-96	0.38	0.23	-0.63	0.08	-0.41	-0.40	-0.51	-0.60	-0.14
g__norank_o__Subgroup_7	0.18	0.32	-0.05	-0.29	-0.46	**-0.51***	**-0.63***	-0.35	0.42
g__Candidatus_*Solibacter*	0.10	0.01	0.05	-0.37	**-0.65***	-0.29	-0.36	-0.22	0.22
g__*Anaeromyxobacter*	0.26	-0.48	-0.15	-0.12	-0.16	0.14	-0.05	**-0.54***	-0.37
g__*Thiobacillus*	-0.04	**0.60***	-0.05	-0.39	-0.37	-0.30	-0.03	0.25	0.37
g__norank_f__*Acidobacteriaceae*__Subgroup_1_	-0.23	0.70	-0.23	0.26	-0.24	-0.51	-0.45	0.32	0.49
g__norank_c__*Bacteroidetes*_vadinHA17	-0.43	-0.12	0.46	-0.35	-0.17	0.17	-0.05	0.16	0.08
g__norank_o__SC-I-84	0.36	0.21	-0.24	-0.31	**-0.56***	**-0.57***	-0.38	**-0.59***	0.34
g__*Bryobacter*	0.14	0.53	-0.16	-0.26	**-0.61***	**-0.78****	**-0.63***	0.11	0.26
g__norank_f__*Nitrosomonadaceae*	0.15	0.56	-0.02	-0.28	-0.38	-0.50	-0.30	0.11	**0.60***
g__unclassified_p__*Chloroflexi*	0.39	0.15	-0.58*	-0.21	**-0.60***	-0.34	-0.41	**-0.57***	-0.26
g__norank_c__OPB35_soil_group	**-0.56***	0.24	0.10	0.45	0.13	-0.03	-0.09	**0.66****	0.43
g__*Anaerolinea*	-0.29	-0.07	-0.38	0.29	-0.01	0.21	0.34	0.03	0.04
g__*Sideroxydans*	0.09	**0.62***	-0.08	-0.41	-0.41	**-0.65***	-0.33	0.16	0.48
g__*Gemmatimonas*	0.29	**0.75***	**-0.56***	-0.19	-0.48	**-0.72***	-0.11	-0.05	0.46
g__unclassified_k__norank_d__Bacteria	0.15	0.50	**-0.63***	0.01	**-0.63***	**-0.63***	**-0.66****	-0.30	0.28
g__norank_c__*Gemmatimonadetes*	0.50	0.02	-0.34	-0.55	**-0.78****	**-0.57***	-0.30	**-0.64****	-0.05
g__unclassified_f__*Gallionellaceae*	-0.17	0.33	0.28	-0.22	-0.16	-0.27	-0.09	0.51	**0.58***
g__Candidatus_*Koribacter*	-0.41	-0.01	0.16	0.38	-0.02	0.09	-0.04	0.41	0.13

g__*Haliangium*	0.27	0.09	-0.41	-0.09	**-0.56***	**-0.55***	**-0.65***	**-0.60***	0.06
g__norank_o__43F-1404R	0.13	0.06	0.28	-0.13	-0.11	-0.42	-0.49*	0.07	0.29
g__*Pseudolabrys*	0.09	0.18	-0.09	-0.41	**-0.59***	-0.58	**-0.67****	-0.29	0.39
g__*Geobacter*	0.04	-0.34	0.22	-0.01	-0.27	0.04	-0.41	-0.24	-0.08
g__*Rhodanobacter*	-0.11	-0.42	0.01	0.07	-0.34	0.18	-0.22	-0.46	-0.11
g__norank_p__*Latescibacteria*	0.32	**0.56***	-0.44	0.12	-0.24	**-0.58***	-0.48	-0.14	0.13
g__norank_c__SJA-15	0.42	-0.12	-0.51	-0.21	-0.33	0.03	-0.25	**-0.64****	-0.29

EC: electricity conductivity; SOC: soil organic carbon; TP: soil total phosphorus; TN: soil total nitrogen; WC: soil water content; DCB-Fe: DCB-extracted iron. Asterisks indicate significant difference (* $0.01 < P \leq 0.05$, ** $0.001 < P \leq 0.01$)

Table S2 Rice yield components in different treatments

Treatment	number of productive ear($\times 10^4$ hm^{-2})	Grain Number Per-panicle	Seed setting rate (%)	1000-grain weight (g)	Actual yield (Kg hm^{-2})
T1	326.1±58.0	95.3±2.8	92.0±4.0	28.4±0.4a	9850±1154.7b
ST0	327.0±34.7	90.2±6.0	91.6±0.3	27.2±0.4b	8350±14.3c
ST1	344.3±37.1	100.5±8.1	93.5±0.9	26.8±1.1b	10650±577.3b
ST2	393.5±68.6	93.2±7.9	93.6±0.7	26.8±0.4b	12133±321.5a
ST3	416.2±50.4	105.9±17.3	90.8±1.0	24.7±0.3c	11100±144.3a

Different letters in the table indicate statistically significant differences among the samples by one-way ANOVA (Tukey-Kramer, $p < 0.05$)
